# Development and Evaluation of Innovation Scales for Mail-Based Human Papillomavirus (HPV) Self-Collection Among U.S. Low-Income Women

**DOI:** 10.3390/nursrep15120449

**Published:** 2025-12-15

**Authors:** Erika Biederman, Katharine Head, Gregory Zimet, Victoria Champion

**Affiliations:** 1 Department of Community Health Systems, Indiana University School of Nursing, Indianapolis, IN 46202, USA; vchampio@iu.edu; 2 Department of Communication Studies, Indiana University-Indianapolis, Indianapolis, IN 46202, USA; headkj@iu.edu; 3 Department of Pediatrics–Adolescent Medicine, Indiana University School of Medicine, Indianapolis, IN 46202, USA; gzimet@iu.edu

**Keywords:** human papillomavirus, cervical cancer screening, self-collection, Diffusion of Innovations, public health nursing

## Abstract

**Background/Objectives**: Low-income compared to high-income women have a higher incidence of and mortality from cervical cancer (CC) due to lower screening rates (under/never-screened). Home-based screening for CC via mailed “self-collection” for human papillomavirus (HPV) testing is an alternative to traditional, provider-collected screening that may be more acceptable to low-income women. Theoretically, adoption of a recent technology, in this case, mailed return of self-collection, is related to the Diffusion of Innovations concepts of advantages and complexity. The purpose of this study was to develop and psychometrically test scales to measure advantages and complexity of self-collection in a low-income, under/never-screened population. **Methods**: Low-income women (n = 168) were recruited in person from food pantries and online using Facebook in the Midwest U.S. After a baseline survey, women were mailed a self-collection kit. We assessed reliability with item analysis and Cronbach’s α and evaluated validity with exploratory factor analysis and t-tests, using mailed kit return as the independent variable. **Results**: Two scales were developed: (1) advantages (Cronbach’s α = 0.84), item–total correlation = 0.51 to 0.69, and (2) complexity (Cronbach’s α = 0.82), item–total correlation = 0.45 to 0.64. Exploratory factor analysis supported items factoring on their respective scales, and *t*-tests supported a relationship between each scale and mailed return of kits. **Conclusions**: Both the advantages and complexity scales demonstrated reliability and validity among low-income women. Future studies should evaluate these scales in a larger, more diverse population. Nurses could use these scales to assess preferences and difficulties associated with self-collection and aid patients with CC screening decision-making.

## 1. Introduction

### 1.1. Background

Globally, cervical cancer (CC) is mostly preventable, yet gaps in routine screening persist across populations who are of low socioeconomic (SES) status [1]. Women who have never been screened or who have been infrequently screened (under-screened) account for most CC cases worldwide [2]. Over 50% of women who develop CC in the United States (U.S.) were not screened in the previous three to five years, i.e., never/under-screeners [3]. Lower CC screening rates [4,5] and higher CC mortality [6,7] are associated with low socioeconomic status (SES). Guidelines for CC screening in the U.S. involve provider-collected Papanicolaou (Pap) testing, human papillomavirus (HPV) testing, or both every three or five years [8]. Recently, the Food and Drug Administration (FDA) authorized self-collected vaginal samples for HPV testing in health care settings [9] and granted de novo authorization for the first at-home self-collection device [10]. Home-based self-collection offers a screening alternative and could increase overall CC screening among low-income (a marker for low-SES) women.

Barriers associated with low SES as well as barriers related to provider-collected screening samples, e.g., time, embarrassment, pain, and fear, prevent many women from adhering to CC screening [11,12]. Mail-based self-collection for HPV testing has the potential to overcome these barriers to provider-based CC screening by allowing women to screen anytime in the privacy of their own homes without the need for a provider or speculum. Studies in the U.S. show that mail-based distribution of self-collection kits is feasible [13,14,15], acceptable [13,16], and preferable compared to Pap testing [17] to low-income women and increases overall CC screening uptake [14,18], thus demonstrating its ability to reach women who are never/under-screeners and low-SES. A limitation of previous self-collection studies is that few theoretical models and instruments related to returning a completed self-collection kit by mail have been developed [13]. Although CC screening as a behavior has been researched for decades [19], self-collection involves different steps and behaviors than a provider-based screening. For example, women may naturally compare self-collection to provider-collection or have difficulties with screening that are specific to self-collection and mailing a test. Reliable and valid instruments are necessary to understand and measure behaviors related to completing and returning mailed HPV testing self-collection kits.

For the current paper, our research hypotheses related to instrument testing included the following: (1) Internal consistency reliability will be supported by a Cronbach’s alpha of 0.7 or better and inter-item correlations of less than 0.7, and (2) validity will be established with items factoring on their respective scales through exploratory factor analysis, and advantages and complexity will be significantly associated with mailed return of self-collection kits in *t*-tests. Our aim was to develop and complete preliminary testing on two scales designed to measure the advantages and complexity of self-collection among low-income women who are outside of CC screening guidelines. These scales will help researchers and clinicians better understand behaviors related to self-collection and can inform the development of interventions to increase overall CC screening.

### 1.2. Theoretical Framework

The Diffusion of Innovations (DOI) Theory was developed by Rogers to study the characteristics of an innovation associated with the uptake of a new technology like self-collection [20]. DOI outlines five perceived attributes of innovations, including relative advantage, complexity, compatibility, trialability, and observability, that influence adoption [20]. Although all five characteristics can affect uptake of new technologies, Rogers suggests that perceived relative advantage is the most important predictor of adoption [20].

Prior qualitative work with stool-based colorectal cancer screening, a similar self-collection cancer screening test, found that two characteristics emerged as important, advantages and complexity [21]. Additionally, although advantage and complexity instruments have not been fully developed in relation to self-collection, items related to these concepts are frequently assessed in self-collection studies such as barriers including privacy and convenience (advantages) as well as unclear instructions and fear of inadequate sampling (complexity) [16,22]. Therefore, these two concepts were chosen for scale development. With respect to the present study, advantages refers to the perceived superiority of self-collection over provider-based Pap/HPV testing [20], whereas complexity addresses how easy the mail-based self-collection kit is to use and understand [20]. Measurement of these concepts could help researchers determine an individual’s preferences and concerns about screening modalities to tailor an intervention to increase screening uptake. Advantages and complexity should be related to the adoption (completion and return) of mailed self-collection for HPV testing.

DOI theory suggests that new health technologies are adopted in a staged process with perceptions of relative advantage and complexity influencing whether individuals move forward through the adoption process or disengage [20,23]. Innovations, in this case, self-collection, are adopted more rapidly when they are perceived as having clear advantages over existing practices, i.e., provider-collected CC screening samples, and low complexity [20]. Determining whether advantages and complexity influence adoption among low-SES women is particularly salient as the personal and structural barriers experienced by these women may decrease perceived advantages and increase perceived complexity of self-collection, limiting its adoption. Low self-collection uptake limits its impact on increasing screening rates in these populations. Thus, measuring advantages and complexity is critical for understanding and predicting adoption of mailed self-collection and designing interventions to enhance screening uptake.

## 2. Materials and Methods

### 2.1. Sample

We conducted a cross-sectional survey study using a mixed convenience sample. We began recruitment of our sample (n = 168) in person but switched to online recruitment with the advent of the coronavirus disease 2019 (COVID-19) pandemic. Inclusion criteria included the following: (1) not up to date (UTD) with 2018 U.S. Preventive Services Task Force (USPSTF) guidelines for CC screening [8], (2) between the ages of 30 and 65, (3) within 200% of federal poverty guidelines [24], and (4) English-speaking only. The lower limit of age 30 for inclusion reflects 2018 USPSTF guidelines for HPV testing at the time of the study, 2019–2020 [8]. Women who had a history of hysterectomy were excluded. Sample size was based on convenience, i.e., how many women responded to the survey; however, for our primary goal of assessing validity, a sample size of n = 168 met the goal for an exploratory factor analysis of having at least five respondents per item [25]. All procedures in this study were approved by the university IRB.

### 2.2. Approach

For in-person recruitment, the first author and a research assistant approached women at two food pantries in Indianapolis, IN from December 2019 until March 2020. Individuals who expressed interest in participating in a CC screening study were given information about the study. Participants were shown a self-collection kit (Dacron Swab with tube, plastic bag, and self-addressed stamped envelope [SASE]) and paper instructions for completing the HPV self-sample. If participants wished to participate, consent was described, and a written consent form provided for signature. Additionally, participants completed a paper Health Insurance Portability and Accountability Act (HIPAA) form that provided the researcher access to their HPV results.

Women completed a baseline paper survey and gave their telephone number, email address, and physical address so they could be mailed a self-collection kit and be contacted with their HPV results if they returned the self-collection kit. Participants also provided a phone number for another person (could provide up to three) so that the researcher could reach them if the participant had positive results and a problem occurred with their personal phone.

Online recruitment involved ads for a CC screening study on Facebook and Craigslist from May 2020 until October 2020. If participants were interested, they were redirected to an eligibility form online in REDCap. Participants who were eligible were then directed to an online consent form with the researcher’s email and phone number for questions. The HIPAA form and survey were also available in REDCap after signing an informed consent form.

Within two weeks of completion of baseline surveys, all participants were mailed a self-collection kit and given a $20 gift card. Women were then called one week after the self-collection kit was mailed to see if they received the kit; if they did not have the kit for any reason, another was mailed. Kits were tracked through the postal service to ensure that they were delivered. Participants had four weeks (after receipt of the kit had been confirmed by postal service) to return the kits before being considered a non-returner.

### 2.3. Measures

Sociodemographic variables were adopted from previous studies. Age was assessed as a continuous variable, and education was divided into those who had a high school degree or less and those who had more than a high school degree. Income was divided into those who made $15,000 or less a year and those who made more than $15,000 a year. Insurance status was categorized as insured vs. not insured. Marital status was divided into married or living with a partner vs. single, separated, divorced, or widowed. Race was assessed as Black, White, Asian, Native Hawaiian or Pacific Islander, American Indian or Alaskan Native, or Other and then categorized into Black, White, and Other due to small numbers in other racial categories. The outcome measure was whether women completed and returned a self-collection kit through the mail (y/n).

Scale items for advantage and complexity were informed by the literature on barriers to Pap testing [26,27], a literature review of acceptance of self-collection [11], instruments used in other self-collection studies [13,18], and a previous study that examined DOI concepts about self-collection tests of other sexually transmitted infections [28]. Our literature review was a targeted, theory-informed review appropriate for early-stage measure development rather than a full systematic review [29]. Searches included backward and forward citation tracing of key self-collection studies and keyword searches, e.g., “self-sampling,” “HPV,” “Pap barriers,” “self-collection acceptability” [30,31]. At the time of our search, we did not identify any validated scales specifically developed for HPV self-collection.

After determining the items to include in the advantages and complexity instruments, four PhD-prepared behavioral science/health communication experts specializing in breast, colorectal, and/or CC screening; HPV testing including self-collection; and HPV vaccination reviewed the items. Minor wording suggestions were offered for individual items. For the advantages scale, one item was suggested to be deleted as it did not capture the conceptual definition of advantages. For the complexity scale, two items were deleted for redundancy. Advantages and complexity were placed on a 5-point Likert scale from strongly disagree to strongly agree.

Seven advantage items were adapted from items related to Pap testing and acceptability of self-collection found in the literature. Items adapted and modified from previous validated barriers scales included Pap testing embarrassment and pain, inability to arrange testing around work and childcare, and not wanting to be weighed on a scale at the clinic [26,27]. Items adapted from self-collection acceptability items included convenience and privacy of self-collection [16]. Our item on preferring self-collection so that they do not have to talk about their sexual history was based on attitudes toward self-collection tests of other sexually transmitted infections [28].

Our nine complexity items were also adapted from instruments used in previous self-collection studies and the literature on self-collection [13,18]. Items adapted from previous instruments included the kit seeming too complicated; not understanding the directions; worry about using the device incorrectly; whether the instructions helped women decide to use HPV self-collection; and words on the instructions being large enough to read [13,18]. Several items related to concerns about mailing the device were adapted from concerns about self-collection of other sexually transmitted infections including the worry that participants would not receive the kit; the mailbox/post office being too far away to easily return the kit; worries about not remembering to return the kit in the mail; and rarely using the mail system [28].

### 2.4. Analyses

All analyses were conducted in IBM Statistical Package for Social Sciences (SPSS) Version 27. Descriptive statistics were analyzed for all sociodemographic variables. Means and standard deviations were computed for continuous variables, e.g., age. Frequencies and percentages were calculated for categorical variables, e.g., education, income, marital status, race, and insurance.

Inter-item correlations and corrected item–total correlations were examined to assess overall reliability. Cronbach’s α coefficients measured internal consistency reliability for both advantages and complexity scales. Missing data were excluded from analysis.

Construct validity was measured through exploratory factor analysis and *t*-tests. First, principal component analysis extraction with varimax rotation was used to extract two factors from all items included on both scales. Factor loadings of 0.4 or greater were considered adequate [32,33,34]. Second, *t*-tests on each scale (advantages and complexity) and the outcome of mailed return (y/n) were performed.

## 3. Results

### 3.1. Sociodemographics and Participant Characteristics

The sample included 168 (aged 30–65) low-income women who consented and completed the survey. In total, 140 women were approached in person; 4 declined and 106 were ineligible, yielding 30 in-person participants. No participants from the in-person recruitment cohort who showed interest in participating in the study and listened to the study information denied consent.

Of the 973 women who completed the online eligibility screening, 140 met eligibility criteria; 2 were excluded due to undeliverable mailing addresses, resulting in 138 participants recruited online. Online participants could choose to stop completing the survey at any time so reasons for non-participation were impossible to collect. Minimal participant data was missing with one–two data points missing per variable.

Table 1 describes the sample demographic characteristics. The average age was 44. Thirty-one percent of the sample were included in the less than $15,000 income group. Sixty percent had more than a high school degree. Most participants (75%) were insured and White (83%). Forty-eight percent were married. Eighty (48%) participants returned kits.

### 3.2. Factor Analysis

We conducted an exploratory factor analysis (EFA) using principal component analysis (PCA) extraction with varimax rotation. Although PCA is technically distinct from common-factor EFA, it is often used in early scale development for identifying item groupings and reducing item redundancy [35]. Prior to extraction, sampling adequacy was assessed. The Kaiser–Meyer–Olkin (KMO) measure indicated sampling adequacy (KMO = 0.833), and Bartlett’s test of sphericity was significant (χ^2^ (120) = 1137.71, *p* < 0.001).

The number of components was fixed to two based on the theorized advantages and complexity constructs. Inspection of the scree plot showed a clear inflection after the second component, supporting the retention of two factors. The two factors explained 33.51% of the variance and represented eigenvalues of 4.69 and 2.21 (both > than 1). The rotated component matrix was examined to determine if items loaded on their scales. Items from the advantages scale loaded on Factor 1 with loadings from 0.57 to 0.75 (>0.4). Items from the complexity scale loaded on Factor 2 with loadings from 0.51 to 0.75 (>0.4). See Table 2 for the factor loading values of each item. Each item loaded on its primary scale and did not load on the other scale at a value above 0.3.

Since the two constructs may have been theoretically related, we also examined a supplementary oblique (Promax) solution. The components showed a modest inverse correlation (r = –0.35), consistent with DOI theory and supporting related but distinct constructs.

### 3.3. Reliability

Item analysis of the seven-item advantages scale was conducted. Items in the correlation matrix were analyzed for high inter-item correlations (>0.7) that indicated unnecessary conceptual overlap between items. Inter-item correlations ranged from 0.34 to 0.72. One item, “Home HPV self-collection is less embarrassing than getting tested by a provider,” was correlated at 0.72 with and conceptually like the item related to privacy. The embarrassment item also appeared to be conceptually like other items related to being weighed and discussing one’s sexual history. Therefore, the embarrassment item was deleted because it did not add unique conceptual information and introduced redundancy. Item analysis conducted without the embarrassment item revealed inter-item correlations ranging from 0.36 to 0.66 and corrected item–total correlations from 0.51 to 0.69. Internal consistency reliability was obtained as a Cronbach’s alpha of 0.84. All values were within acceptable ranges [36]. The mean for the final advantages scale was 24.22 (standard deviation [SD] = 4.06). The corrected item–total correlations and item means for the final advantages scale are shown in Table 2.

Next, item analysis for the 9-item complexity scale was conducted. Inter-item correlations in the correlation matrix ranged from 0.18 to 0.71. One item, “The words on the instructions were not large enough to read”, was highly correlated at 0.71 with the item about the instructions helping them decide to use the HPV self-sampling kit. Conceptually, the “font size” item reflected a formatting/health-literacy concern rather than procedural complexity, as defined by DOI. Therefore, the “words not large enough to read” item was deleted because it lacked clear conceptual alignment with the construct and contributed redundancy. Inter-item correlations after deletion of the “words not large enough to read” item ranged from 0.18 to 0.67. Corrected item–total correlations ranged from 0.45 to 0.64. Cronbach’s alpha was 0.82. The mean for the final complexity scale was 15.71 (SD = 4.44). The corrected item–total correlations and item means for the final complexity scale are shown in Table 2.

### 3.4. Validity

Missing data on scale items were <1% per item; listwise deletion was applied for the *t*-tests. *t*-tests indicated a significant difference (*p* = 0.016) in the expected direction in the mean of the advantages scale between those who returned self-collection vs. those who did not (mean [M] = 25.01 vs. 23.50, t(166)] = –2.45). The mean difference of –1.51 had a 95% confidence interval (CI) of –2.73 to –0.29. The effect size was in the small–medium range (Cohen’s d = –0.38, 95% CI [–0.68, –0.07]).

Similarly, a *t*-test indicated a significant difference (*p* = 0.043) in the expected direction in mean of complexity between returners vs. non-returners (M = 16.36 vs. 14.97, t(165)] = 2.04). The mean difference of 1.39 had a 95% CI of 0.04 to 2.74. The effect size was small but meaningful (Cohen’s d = 0.32, 95% CI [0.01, 0.62]).

## 4. Discussion

Previous research indicates that innovation attributes, specifically, advantages and complexity, are related to adoption of a new technology such as self-collection [37,38]. In this study, we assessed the reliability and validity of scales capturing these constructs among low-income, under/never-screened women. One item was removed from each scale to improve item correlations and overall reliability. After removing these items, all scales demonstrated high internal consistency. Exploratory factor analysis demonstrated validity with items factoring onto their respective scales with loadings greater than 0.4. Validity was further demonstrated by differences in the means of each scale between those who were returners and non-returners of self-collection in *t*-tests. Our results indicate that both advantages and complexity were related to mailed return of self-collection kits in the expected direction. As predicted by DOI, both higher advantages and lower complexity scores were associated with mailed return of kits.

This is one of the first studies to develop instruments related to the behavior of returning a self-collection sample via mail. Previous self-collection studies have typically measured kit return using single items or brief, study-specific questionnaires rather than using validated, theory-based scales [15,39,40,41]. A prior review examined self-collection acceptability along multiple dimensions and found that most women report self-collection as not embarrassing (91%), private (88%), and easy to use (91%) [39] but did not report these as a part of validated instruments. Establishing measures with a theoretical framework is an essential step in the development of effective behavioral interventions [42,43]. Ideally, self-collection will reach women who are under/never-screened due to barriers associated with provider-based Pap/HPV testing [15,44,45]. Although self-collection has recently been approved as a screening method in the U.S. [9,10], low uptake of self-collection could limit its population-level impact. Advantages and complexity could enhance implementation outcomes such as acceptability, adoption, and return of self-collection kits [46]. For example, a home-based colorectal cancer screening option that is conceptually like HPV self-collection is the fecal immunochemical test (FIT). In a previous study, advantages of a pharmacy-based FIT program were positively associated with willingness to use FIT, similar to our finding that higher perceived advantages were related to mailed return of HPV self-collection kits [47].

As self-collection becomes part of U.S. screening guidelines, provider-based screening remains as an option. Most women will either have experience with or are aware of provider-based Pap/HPV testing and may compare it to self-collection [15]. Our advantages scale provides a way to assess how the two screening modalities are compared by determining who prefers self-collection vs. provider-based screening. Previous colorectal cancer screening research shows that tailoring to test preference, i.e., stool testing vs. colonoscopy, has resulted in higher overall colorectal cancer screening uptake [48,49]. As self-collection becomes more widely available in the U.S., programs could similarly tailor messaging to women’s preferred CC screening modality. Previous studies of other screening tests, including mammography, colorectal cancer screening, and cervical cancer screening, have incorporated tailored messages into video- or web-based interventions, with the message content determined by participants’ responses [50,51]. For example, if a woman selects “I don’t have time to see a provider” as a barrier to screening, the intervention delivers a tailored message that reinforces the importance of prioritizing her health [52]. Similarly, women who score higher on the advantages scale could be targeted with messages about the advantages of self-collection in a tailored intervention. For example, if a woman scores highly on the convenience of self-collection, a researcher could highlight how the sample can be completed at home in one’s own bathroom anytime without having to make or drive to an appointment. If a woman scores lower on the advantages scale, it may indicate that she is less likely to return a self-collection kit or prefers provider-based testing, so she could be directed to her preferred screening modality.

Our complexity scale could be used to assess women who may need more assistance with self-collection in an intervention. For example, if a woman scores highly on the item about remembering to return the kit in the mail, she may need reminders, e.g., letters, phone calls, or text messages, to prompt her to return the kit. Reminders have consistently increased completion of mailed FIT kits [53] and HPV self-collection kits [54] in prior studies. While participants were given SASE, some individuals may face structural barriers related to neighborhood mailbox access that may affect kit return. Mailed FIT programs sometimes report patient concerns about the postal service, i.e., the sample will be lost, so these programs often allow kit return at a clinic/lab as well as through mail, which demonstrates the need for multiple return options when mail access is limited/inconvenient [55,56]. In our sample, women who scored highly in the item related to mailing their kit back, e.g., lack of convenient mailbox access, may benefit from alternative return options. Women who have difficulty understanding the self-collection instructions may benefit from extra support such as a patient navigator/nurse talking them through the procedure or video instructions, as has been reported in mailed FIT programs [56,57].

In addition to informing intervention and implementation research, these scales have practical implications for public health nursing, community screening programs and policy. Public health nurses and clinic-based patient navigators could use the advantages and complexity scales to identify women who may benefit from tailored counseling, reminders to support kit completion, and/or being counseled on their preferred screening method (self-sampling vs. provider sampling) [48,52,58]. One recent study found that a patient navigation group had higher overall CC screening (including both self-collected and provider-collected samples) participation compared to a group that only received a telephone reminder [58]. Tailored phone navigation significantly increased cervical, breast, and colorectal cancer screening uptake compared to usual care in another study on U.S. rural clinics [52,59]. Screening programs such as the Centers for Disease Control and Prevention (CDC) National Breast and Cervical Cancer Early Detection Program which serve low-SES populations could integrate these measures into their workflows, like other evidence-based practices, to increase screening uptake [60]. At the policy level, validated measures of perceived advantages and complexity can guide equitable guideline implementation of self-collection. For example, if certain clinics or populations have higher complexity scores, this could guide state or federal funding for patient navigation or other interventions to these areas [60]. Additionally, if these scales were used widely by clinics, complexity scores could be compared to ensure that there are no disparities between population groups or geographic regions.

## 5. Limitations

First, these scales were validated among low-income, under/never-screened women living in Indiana who had internet and Facebook access. As such, findings may not be generalizable to women with different socioeconomic, geographic, or access characteristics. However, a recent poll indicates that virtually all women aged 30–49 and 85% of women aged 50–64 have access to a smartphone, tablet, or computer with internet access [61], supporting the ability to reach underserved women through online recruitment. Additionally, the sample size was small, reflecting preliminary and exploratory scale development. Future studies should conduct confirmatory factor analysis (CFA) in larger and more diverse populations to cross-validate the factor structure and apply stricter item-retention criteria (e.g., standardized loadings ≥ 0.50) across subgroups.

Second, perceptions of privacy, convenience, and discomfort related to self-collection may vary across cultural, linguistic, and geographic contexts. While not measured in the current study, factors such as cultural norms related to bodily autonomy [62], modesty [63], or medical mistrust [64] may influence how women interpret and respond to specific items. Control over the screening process and health care decisions may be particularly important to self-collection acceptability and adoption [65]. Therefore, cross-cultural adaptation and measurement equivalence testing, e.g., multi-group CFA, are necessary to ensure that these instruments reflect different racial/ethnic groups, languages, and health-system environments. In addition, online recruitment required participants to have internet access and a stable residential address to mail the kit. Thus, women experiencing homelessness or unstable housing may have been underrepresented. Future research should incorporate alternative kit distribution and return methods to reach women without reliable internet or mailing access.

Third, other implementation factors that could affect adoption such as device type and cost were not assessed [66]. Our study used Dacron swabs for self-collection; however, a recent FDA-approved device for self-collection differs from Dacron swabs so these scales may need to be reevaluated with the FDA-approved Teal device or any other devices approved in the future [10]. Cost considerations were not assessed but may influence willingness to use self-collection compared to free or low-cost Pap tests available through national screening programs. Future research should examine financial and insurance-related factors that may shape preferences and adoption of self-collection.

## 6. Conclusions

The current study provides preliminary instrument testing of advantages and complexity scales. These instruments were found to be reliable and valid for assessing adoption of mail-based HPV self-collection among low-income, under/never-screened women. Advantages and complexity scales could help researchers and clinicians identify women who may prefer self-collection and/or need additional support with self-collection. These measures could be used for tailoring in intervention development. Future work should validate these instruments in larger and more diverse populations and evaluate their usefulness in implementation of self-collection in clinics and public health settings as this screening modality becomes more widely available.

## Figures and Tables

**Table 1:** Demographic characteristics. (n = 168).

Sociodemographics	n or M	% or SD
Age (M, SD)	44.01	9.82
Education (n, %)		
High school degree or less	66	39.8
More than a high school degree	100	60.2
Income (n, %)		
Less than $15,000 a year	51	30.5
More than $15,000 a year	116	60.5
Insurance status (n, %)		
Insured	125	75.3
Uninsured	41	24.7
Marital Status (n, %)		
Married or partner	87	52.4
Divorced, widowed, separated, or single	79	47.6
Race (n, %)		
Black	29	17.4
White	124	74.3
Other ^1^	14	8.4

Abbreviations: M, mean; n, number in sample; SD, standard deviation. ^1^ Asian = 2, Native Hawaiian, Pacific Islander = 1, American Indian/Alaskan Native = 4, Other = 7.

**Table 2:** Corrected item–total correlation coefficients, item means, and item loadings for exploratory factor analysis.

Item	Correlation	X^−^	Factor Loading
Advantages			
Home HPV self-collection is more private than going to a clinic or provider’s office for a Pap/HPV test.	0.61	4.36	0.73
HPV self-collection is more convenient than a provider collected Pap/HPV test	0.69	4.32	0.77
HPV self-collection is less painful than a Pap/HPV test.	0.51	3.63	0.57
HPV self-collection is easier to arrange around work or childcare than a provider collected Pap/HPV test.	0.64	4.28	0.72
HPV self-collection allows me to not have to be weighed at the clinic/office unlike a provider collected Pap/HPV test.	0.67	3.88	0.75
HPV self-collection allows me to avoid talking about my sexual history unlike a provider collected Pap/HPV test.	0.60	3.76	0.70
Complexity			
The HPV self-collection kit seemed too complicated.	0.64	2.09	0.75
I can’t understand the directions to collect the HPV sample.	0.51	2.06	0.66
I am worried that I will use the HPV self-collection device incorrectly.	0.60	2.19	0.72
The instructions did not help me decide whether to use the HPV self-collection device.	0.63	2.11	0.74
I am worried that I will not receive the HPV self-collection kit in the mail.	0.45	2.27	0.58
A mailbox or post office is too far away for me to easily return the HPV self-collection kit.	0.63	1.65	0.70
I am worried I won’t remember to return the kit in the mail.	0.52	1.70	0.63
I have rarely or never used the mail system.	0.44	1.55	0.51

## Data Availability

The full dataset cannot be shared publicly because of potentially sensitive information disclosed by participants. The deidentified data underlying the results presented in the study are available from Erika Biederman, at emcconne@iu.edu.

## Public Involvement Statement

No public involvement in any aspect of this research other than as participants.

## Guidelines and Standards Statement

This manuscript was drafted against the STROBE checklist for cross-sectional studies research.

## Abbreviations

The following abbreviations are used in this manuscript:

CC	Cervical cancer
HPV	Human papillomavirus
USPSTF	United States Preventive Services Task Force
UTD	Up to date
FIT	Fecal immunochemical test
SASE	Self-addressed stamped envelope
EFA	Exploratory factor analysis
CFA	Confirmatory factor analysis
DOI	Diffusion of Innovations
